# Variation of skeletal muscle ultrasound imaging intensity in horses after treadmill exercise: a proof of concept for glycogen content estimation

**DOI:** 10.1186/s12917-021-02818-9

**Published:** 2021-03-16

**Authors:** Sarah A. Tabozzi, Giovanni Stancari, Enrica Zucca, Michela Tajoli, Luca Stucchi, Claudio L. Lafortuna, Francesco Ferrucci

**Affiliations:** 1 Present Address: Croce Rossa Italiana, Comitato Nazionale, Via Ramazzini 37, Roma, Italy; 2 Laboratorio di Medicina Sportiva del Cavallo “Franco Tradati”, Università degli Studi di Milano, Lodi, Italy; 3 Istituto di Fisiologia Clinica, CNR, Sede di Milano, Milano, Italy

**Keywords:** Ultrasound, Greyscale, Exercise, Treadmill, Muscle glycogen, Horses

## Abstract

**Background:**

Glycogen in skeletal muscle is a major source of energy during exercise and an important determinant of endurance capacity, so that its measurement may provide a meaningful marker of athletes’ preparation and a possible predictor of performance, both in humans and in equines. Gold standard of glycogen concentration measurement is the histochemical and biochemical analysis of biopsy-derived muscle tissue, an invasive and potentially injuring procedure. Recently, high-frequency ultrasound (US) technology is being exploited in human sports medicine to estimate muscle glycogen content. Therefore, aim of the present study is to evaluate the feasibility of US assessment of muscle glycogen in equines.

**Results:**

US images of *gluteus medius* (GL) and *semitendinosus* (ST) muscles were obtained on eight healthy horses (3–10 years) before and after a steady-state exercise on treadmill (velocity: 4.0–12.5 m/s; duration: 2–20 min; heart rate: 137–218 b/min). Average image greyscale intensity was significantly different between GL and ST, both before and after exercise (*p* < 0.001). Comparing baseline and post-exercise US images, significant increase in greyscale intensity has been observed in ST (*p* < 0.001), but not in GL (*p* = 0.129). The volume of the exercise was significantly correlated with exercise-dependent change in image intensity (R^2^ = 0.891), consistent with a reduction of glycogen muscle stores resulting from aerobic activity.

**Conclusions:**

US technique evidences also in horses muscle changes possibly associated to glycogen utilisation during exercise. Present results on a small sample need to be further confirmed and provide preliminary data warranting future validation by direct glycogen measurement through biopsy technique.

## Background

The horse is a versatile and extraordinary athlete, as it can be appreciated in the variety of existing equine sport disciplines. Horses, regardless of their origin and their aptitude, have in common the ability to perform physical activities, including jumping and running, at a level of performance that surpasses most other similarly sized animals, thanks to their superior specific aerobic capacity [13]. This is due to a number of physiological adaptations, mostly independent of training, including alveolo-capillary features, cardiac size, haemoglobin concentration and musculoskeletal characteristics [2, 5, 11, 13]. Moreover, beside the capabilities of the machinery for the delivery of O_2_ throughout the entire path from the alveolar gas to mitochondria, also the availability of energy stores of substrates in skeletal muscle tissue (i.e. glycogen concentration) is recognised to impact on athletic endurance performance. The concentration of glycogen in muscles is reported to be higher in horses, compared to humans and other mammals, being 550–600 mmol/kg dry weight in equine muscle, as compared with 300–400 mmol/kg dry weight in human muscle (Snow et al. 1991).

During vigorous exercise, as in the case of most of competitive equine activities, the blood-derived glucose can guarantee less than 10% of the energy used [14]. The presence of adequate substrate deposits in the muscle cell allows the horse to sustain prolonged exercise. It has been shown that skeletal muscle glycogen depletion is associated with a reduction of exercise capacity and a significant lengthening of recovery times [15]. Since muscle glycogen content is modifiable by adjustments of appropriate dietary regimen in relation with training protocols, the measurement of muscular reserves is a key point in the management of the athlete horse.

At present, the gold-standard measure of muscle glycogen concentration is considered to be achieved by histochemical analysis of percutaneous biopsy samples, generally performed on the *gluteus medius*. Major limitations of biopsy assessment are the invasiveness and the traumatic action of the procedure: although a single biopsy allows the regeneration of myofibrillar tissue, repeated biopsies with an interval less than 7 weeks or affecting the basement membrane can prevent a complete repair of the muscle and cause proliferation of granulation tissue, with a keloid formation [17]. This represents a major drawback of the measurement of glycogen storage in sport environment, optimally requiring multiple sampling during training and/or pre-post assessment in the occasion of competitions.

Less invasive techniques have been scarcely applied in veterinary medicine. However, clinical studies in human sports medicine validated the use of muscular ultrasound (US) as a possible alternative to muscle glycogen measurement through chemical analysis of biopsy samples [7, 9, 18].

Based on the osmotic properties of glycogen molecule, binding 3–4 g of water for each gram of glycogen [19], the technique has provided the estimate of glycogen availability by assessing the average US intensity on images of human quadriceps, sampled on standardised position, pre and post exercise. High concentration of glycogen (and water) in baseline images is associated to lower average US intensity (darker), while, due to glycogen depletion and the consequent loss water, image intensity shifts towards hyperechoic US intensity (clearer). Results have been confirmed, using biopsies, to quantitatively describe variations in the glycogen content after a relevant depleting exercise [18].

The current study aims to evaluate the use of this technique in equines, in order to explore a non-invasive method, alternative to muscle biopsy, and to provide preliminary background data for future validation studies assessing skeletal muscle glycogen concentration in horses with US technique.

## Results

**Fig. 1:**
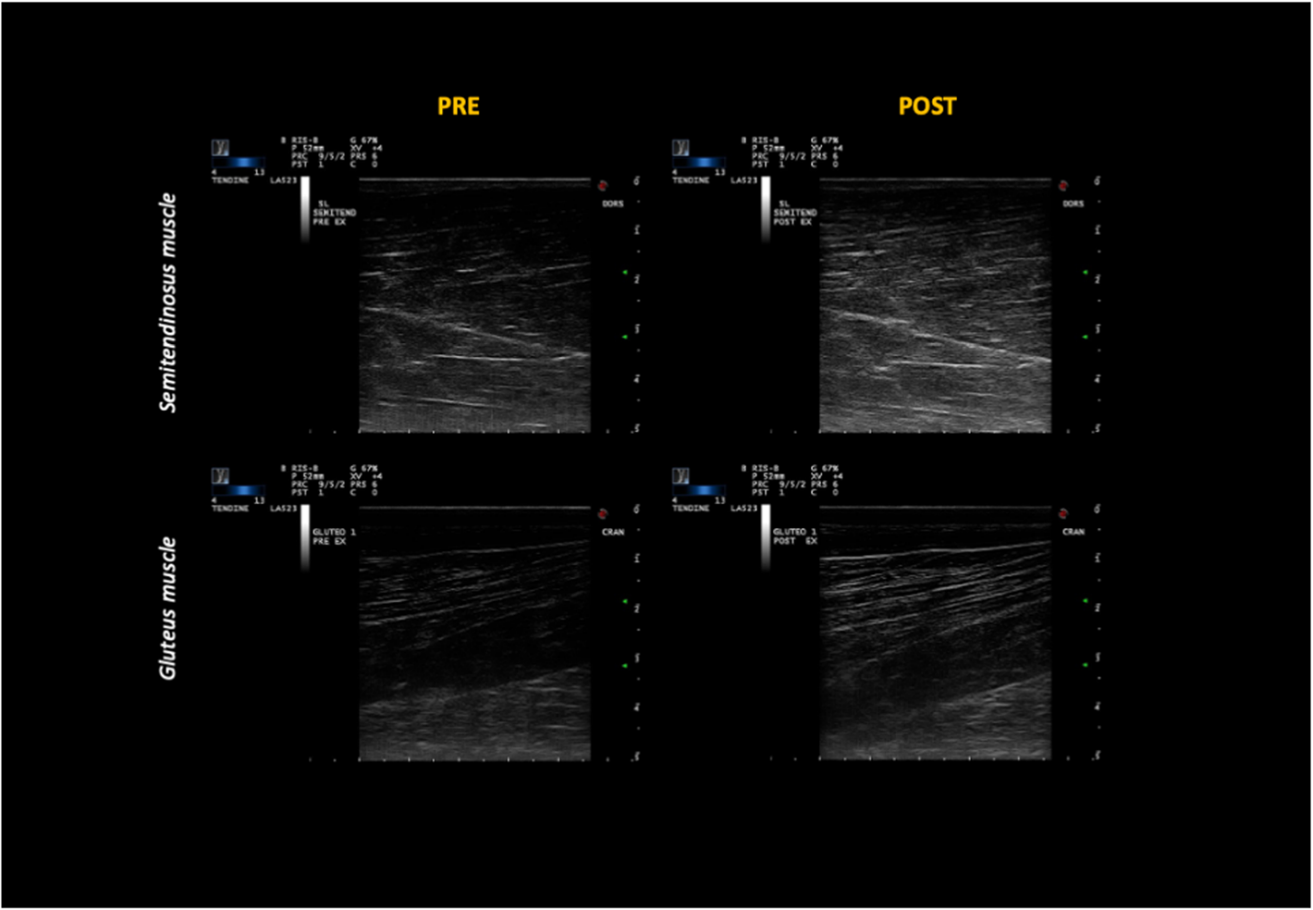
Qualitative ultrasound images of *semitendinosus* (ST, upper panels) and *gluteus medius* (GL, lower panels), acquired before (PRE, left panels) and after (POST, right panels) exercise in the same anatomical area. as confirmed by the structural landmarks visible in the images

Figure 1 shows US images relative to ST and GL muscles, acquired before and after exercise in the same anatomical area, as confirmed by the structural landmarks visible in the images. For each muscle, a qualitative difference in US intensity between the two conditions (before and after exercise) can be appreciated, though more evidently for ST muscle.

**Fig. 2:**
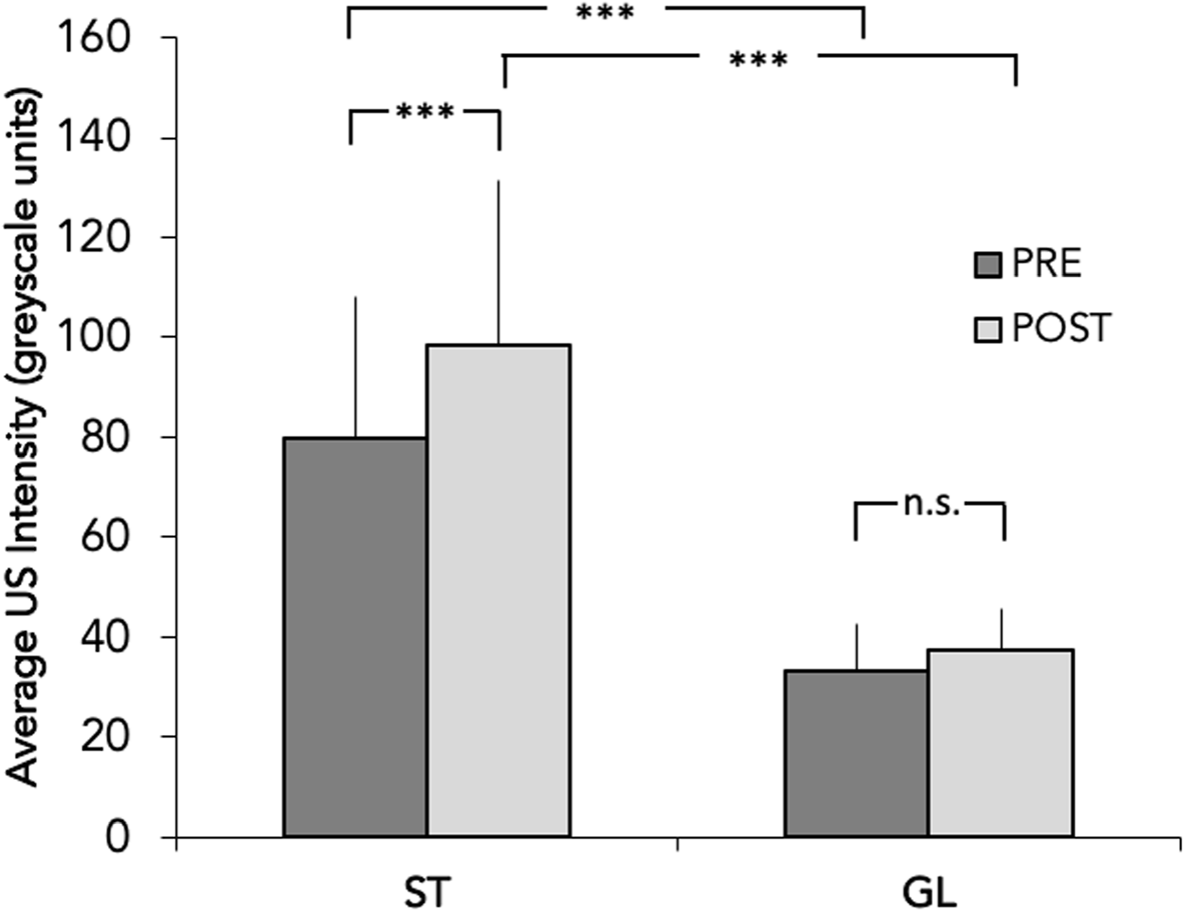
Average value of ultrasound intensity, in greyscale units, measured in *semitendinosus* (ST) and *gluteus medius* (GL) of all horses before (darker bars) and after (lighter bars) exercise. Significance of difference between muscles and between conditions are assessed with a Student’s t-test for paired measurements (***: *p* < 0.001)

The quantitative comparison of US images is presented in Fig. 2, which shows the average value of US intensity, in greyscale units, measured in all horses before and after exercise. Student’s t-test for paired measurements indicated that both before and after exercise average US intensity is higher in ST muscle than in GL muscle, whereas the same paired test showed that a statistically significant difference between the values observed before and after exercise was found for ST muscle but not for GL muscle.

**Fig. 3:**
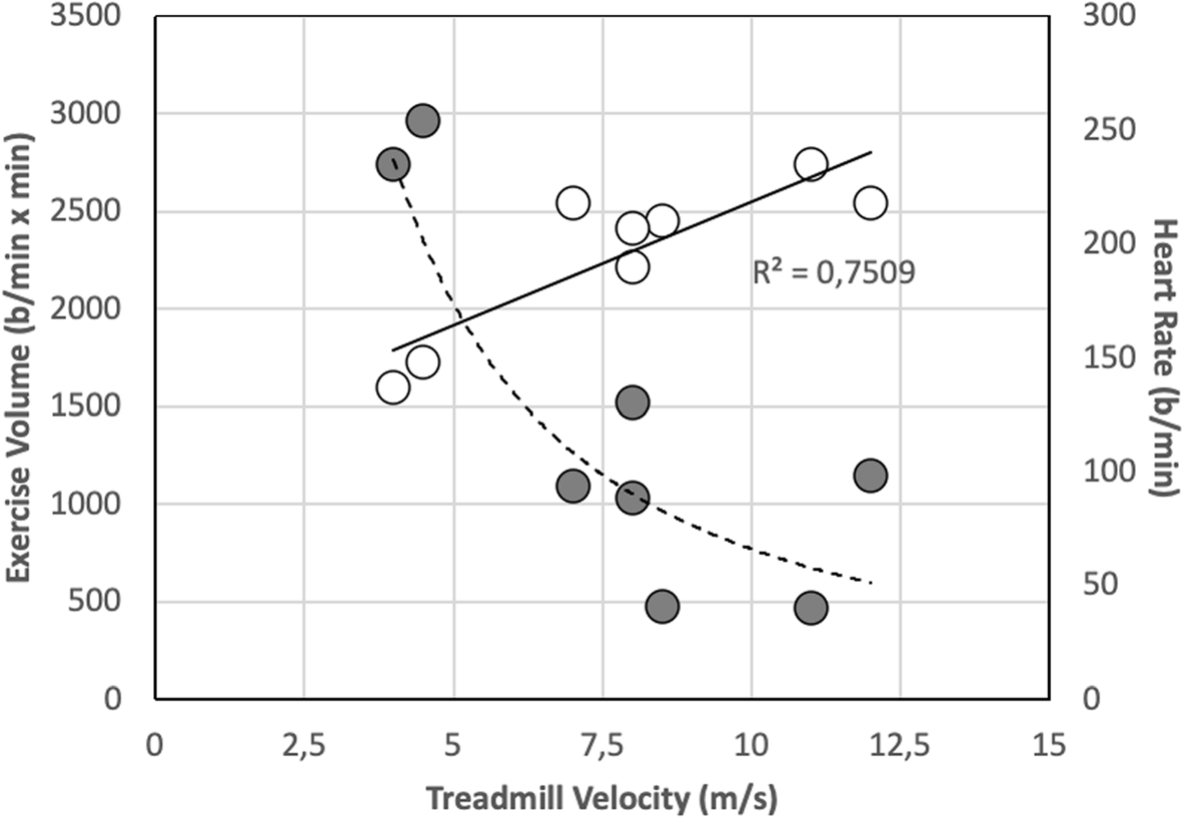
Volume of exercise performed during the steady-state test (left axis) and attained heart rate (right axis) are plotted as a function of treadmill velocity

Figure 3 shows that volume of exercise, as indirectly estimated using the combination of velocity and duration of the tests, is a decreasing function of treadmill velocity, whereas the average cardio-dynamic response observed throughout the steady-state exercise is a linear positive function of velocity.

**Fig. 4:**
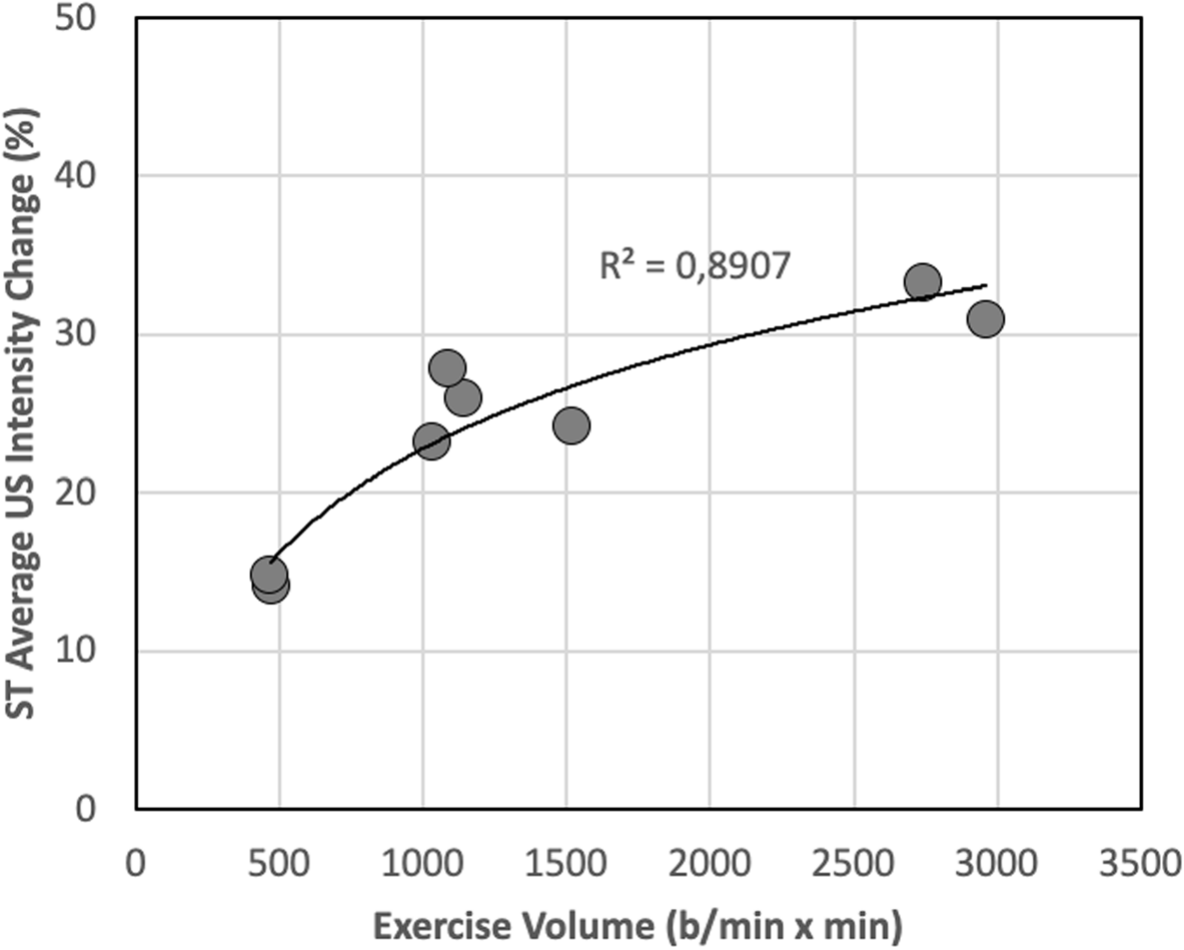
The change of ultrasound intensity displayed by *semitendinosus* muscle in individual horses after exercise, expressed as a percent of the pre-exercise value, is plotted as a function of the volume of exercise sustained during the test on treadmill

As shown in Fig. 4, the change of US intensity displayed by ST muscle in individual horses after exercise, expressed as a percent of the pre-exercise value, resulted significantly correlated with the volume of exercise sustained during the test on treadmill with a positive curvilinear trend.

## Discussion

The current study shows that significant changes proportional to treadmill exercise of different intensities and duration, can be detected in US image greyscale intensity of ST muscle in horses and can be interpreted as the decrease of intramuscular glycogen stores, due to their metabolic utilisation during exercise.

Ultrasonography has been introduced in equine medicine in the early 1980s. At present, in the domain of locomotor system, US represents a common veterinary tool for the diagnosis, evaluation and monitoring in the diseases of tendinous, ligamentous and muscular structures [6]. Application of US imaging for muscle glycogen assessment in horses has not been studied before. In humans, the development and validation of US muscle imaging technique for the study of muscle glycogen contrasted with biopsy sampling has been described for the first time by Hill and Millán (9) and further confirmed by Nieman et al. [18]. Quadriceps muscle US image intensity before the race has been found to be a significant predictor of athletic performance in ultra-marathon runners (personal observation, submitted for publication).

Indeed, glycogen stores in muscle provide the principal energy source in prolonged exercise, so that endurance exercise results to induce marked glycogen depletion and to bring human athletes to exhaustion [1]. Also in horses, glycogen depletion is considered a limiting factor to the athletic performance during prolonged physical activity [10].

Indeed, overall glycogen storage capacity and post exercise levels are reported to vary individually, in horses as well as in humans, depending on training and dietary regimens [8, 20, 24]. Yet, we found in the same individual a significantly different US intensity between GL and ST muscle (see Fig. 2), a finding possibly deriving from a systematic lower glycogen content of ST, due to a different fibre composition among these muscles. In fact, fibre composition of GL and ST was studied in Thoroughbred horses [12] and it was found that Type IIa fibres, which are characterised by the highest glycogen content, account for the 42% of GL, and 26% of ST, thus suggesting that a different fibre composition may induce a difference in glycogen content detectable through US imaging.

Although it cannot be excluded that such a different fibre composition in GL and ST might also be due to a different biomechanical role played by these muscles during locomotory activity, Crook et al. [3] could not detect any significant difference in muscle electromyographic activation among hip extensor muscles during equine walk and trot at different velocities and inclines on treadmill. However, it cannot be excluded that part of the overall variability in US intensity measured after exercise in the considered muscles may derive from individual differences in biomechanical (and hence metabolic) muscle involvement deriving from the gait used during the exercise test (trot or canter).

Given the notion that GL muscle has been reported to be fairly more abundant in glycogen rich IIa fibres, it is surprising that we could not detect changes after exercise in its US features associated with glycogen content, at difference with ST muscle (see Fig. 2). Although a linear correlation was reported in human athletes between the specific glycogen content of *vastus lateralis* muscle (assessed by chemical analysis of specimens obtained through muscle biopsy) and US scores and between their change after exercise exhaustion ([9]; Niemanet al. 2015), a comparison between different muscles is not available. Moreover, horses’ muscles are reported to have a considerably higher glycogen content than that of humans [21] and GL in particular may have a glycogen concentration out of the range of the explored linearity in the correlation between muscle glycogen content and US imaging found in humans. With very high glycogen concentrations, the correspondingly more elevated water content in tissues may interfere with US imaging (lower greyscale intensities and darker images) and prevent an accurate and proportional detection of glycogen. This is a serious issue, potentially limiting the use of US in horses and requiring a further detailed clarification.

However, present results show that US grayscale intensity displayed by ST muscle is significantly affected by exercise. Moreover, the muscle US changes after steady-state exercise bouts are indeed proportional to the volume of performed activity (see Figs. 2 and 4), considered as the product of average HR attained throughout the test multiplied by the duration of the test, and may reflect the expected decrease of skeletal muscle glycogen stores induced by the aerobic activity sustained during exercise. Due to the linear correlation between HR and V’O_2_ observed in horses during locomotion on treadmill [4], the so-calculated volume of exercise directly correlates with the overall amount of O_2_ utilised during the exercise test and with glucose therein oxidised as energy substrate, neglecting the shift between carbohydrate and fats utilisation at the different intensities of exercise. Thus, the amount of muscle glycogen reduction, as supposedly represented by the changes in US muscle features, seems to closely match the metabolic demand imposed by exercise. So, it appears that, during a single continuous session of aerobic activity, the overall balance of glycogen depletion is dominated by the total volume of exercise more than the absolute intensity of exercise: during sessions of near to maximal exercise, due to the low sustainability, the percentage of total glycogen used is relatively lower, compared to a more protracted exercise of lower intensity, when a substantial depletion of total deposits ensues, albeit at a slower rate [16, 22, 23].

It is interesting to remark that a certain degree of alinearity in the relation between US intensity change and exercise volume can be appreciated in Fig. 4. Since, in the present exercise protocol, small volumes of exercise correspond to very short and intense exercise bouts, a smaller change in US intensity (and hence glycogen utilisation) during small volumes of exercise may be hypothesised to result from a high contribution of anaerobic energy sources sustaining these intense and short exercise bouts.

## Conclusions

In conclusion, we observed significant changes in US greyscale intensity in ST muscle imaging after exercise, proportional to the energy requirements of the exercise volume. We postulate that these changes qualitatively correspond to the reduction of muscle glycogen stores induced by the energy demand of exercise. Since, also in horses, the use of ultrasound technology may offer a low-cost, non-invasive and simple alternative to histochemical analysis through muscle biopsy for glycogen assessment, these results are promising.

However, due to the limited number of horses and tested muscles and absence of validation against muscle biopsy in equines, some caution should be used in interpreting present results, and further investigation seems warranted in the future to validate the technique also in horses.

## Methods

*Gluteus medius* muscle (GL) and *semitendinosus* muscle (ST) have been investigated with US scan in 8 healthy horses, before and after treadmill exercise**.**

### Subjects

Eight private horses (Thoroughbreds (*N* = 3), Standardbreds (*N* = 3), Hackney ponies (*N* = 2); 3 females, 3 males and 2 geldings; 3–10 years old; mean body mass: 452 ± 113 kg) took part into the experiments. All horses were actively in training as racehorses (*N* = 5), show jumping (N = 2) or combined driving (*N* = 1). The horses had been admitted to the Equine Medicine Unit of the Veterinary University Hospital as outpatients for a performance profiling test. All the horses admitted to the study had no history of rhabdomyolysis syndrome and did not show an increase of muscular enzymes after exercise tests. Before the day of the experiment, horses had been housed in the facilities of the laboratory for 3 days and were fed with concentrate (2–4 kg/day) and hay ad libitum.

The protocol of the present study was a part of the clinical procedures for the functional evaluation of exercise performance which are in allowance with the institutional ethical standards for veterinary diagnostics. All horses’ owners gave a written informed consent.

### US image acquisition and analysis

All image acquisitions were performed while the horse was restrained in crossties in the clinical standing stock and stand squarely with equal weight on all four limbs, on a firm, level ground surface. The appropriate sites for US imaging were chosen using anatomical landmarks. Specifically, for GL, first a line was drawn between the sacral tuber to the tuber coxae. Then, starting from the middle of this line another line was drawn to the ischiatic tuberosity. The site for the ultrasonographic scan was identified at the cranial 1/3 of the latter line. Instead, for ST a line was drawn from the ischiatic tuberosity perpendicular to the ground, and the site was chosen at 1/3 of the distance between the ischiatic tuberosity and the cutaneous area corresponding to the distal portion of ST. Two rectangular shaped shaves, corresponding to transducer surface, were performed on the coat of the horses in the area corresponding to the sites for the ultrasonographic scan of GL and ST muscles. This procedure avoided possible different positioning of the probe before and after exercise.

The acquisitions were performed by a single operator, using default settings of brightness, contrast and image depth for visualization, with a 12 MHz linear transducer and a standard diagnostic high-resolution ultrasound system (ClassC Esaote, Italy). All the settings were maintained during the scan procedures before and after the treadmill exercise.

The transducer was oriented longitudinally to the muscle and ultrasound gel was applied on the probe. During acquisition pressure applied to the probe was minimal, to avoid any undue distortion of tissue under analysis. The same procedure was repeated for acquisition of images within 30 min after the termination of the exercise test.

Three images for each muscle were acquired in DICOM format, both before and after treadmill exercise, and were analysed using the public domain Java image processing program, named ImageJ. A trained operator manually selected for each image a region of interest, including all the visible part of the analysed muscle, excluding superficial and deep fasciae and calculated average intensity of selected area on a 0–254 greyscale. Scores obtained on the three images of the same muscle were averaged for each condition. Repeatability of measurement with probe repositioning was previously tested on nine consecutive images of the same muscle and yielded a coefficient of variation of 1.992%.

### Exercise test

**Fig. 5:**
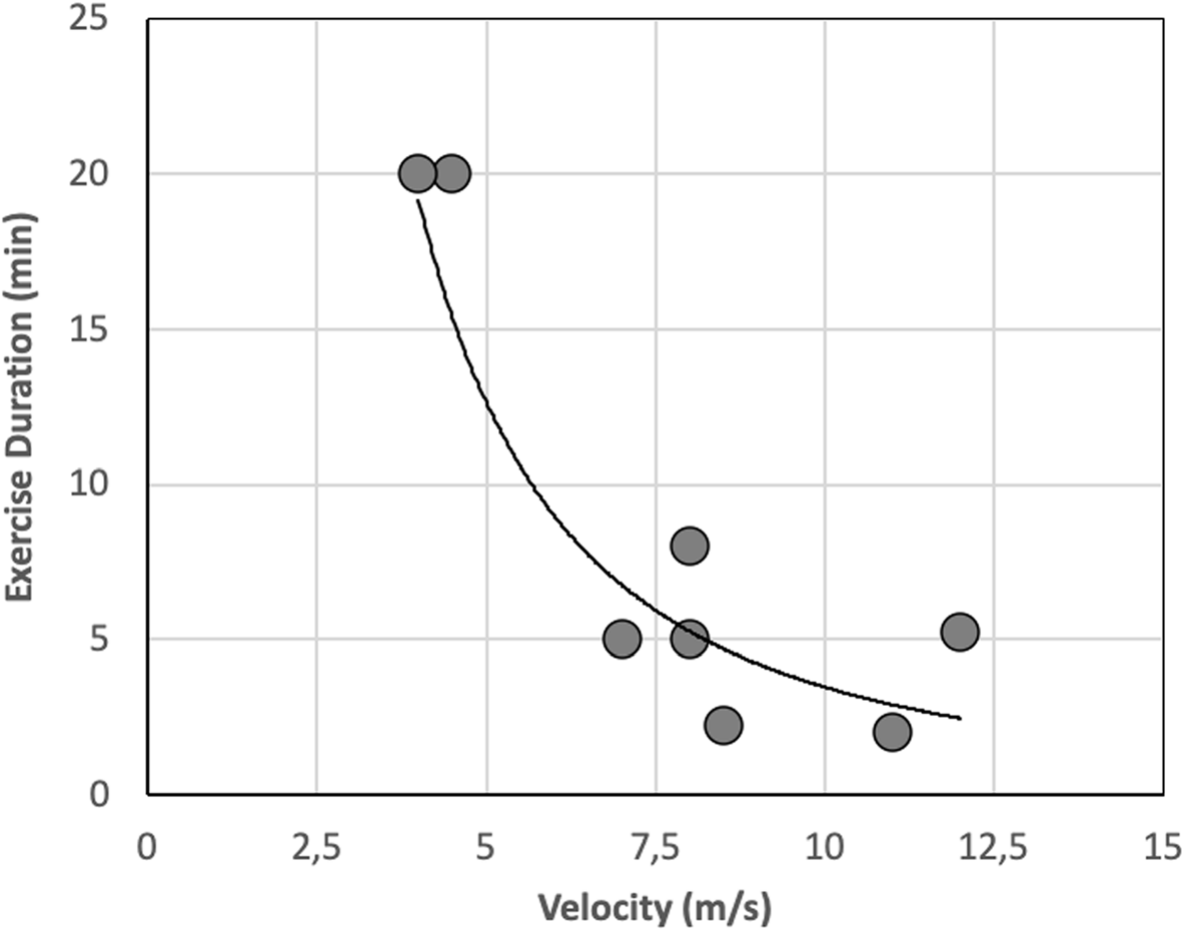
The relationship between duration of exertion and treadmill velocity performed during the steady-state exercise by the different horses participating to the experiment

All horses exercised on high speed treadmill (Sӓto I, SATO, Sweden) on flat. After a 7-min warm up (4 min pacing at 1.5 m/s and 3 min trotting at 4.0 m/s) horses performed a test in steady-state conditions with a combination of velocity and duration appropriate for the individual level of physical conditioning, in a range of velocities comprised between 4.0 and 12 m/s, with a duration ranging from 2 to 20 min. The combination of duration of test and treadmill velocity performed during the steady-state exercise by the different horses participating to the experiment is presented in Fig. 5, which shows that duration of exertion is a decreasing function of treadmill velocity. Throughout the entire duration of treadmill exercise, heart rate (HR) was continuously monitored and recorded by means of a wearable meter (Polar Equine heart rate monitor, M400).

Since, during exercise, HR is a linear function of the corresponding oxygen consumption (V’O_2_), the volume of exercise performed during the test was estimated by the product of the average HR during the steady-state period times the duration of this exertion, which represents the total amount of energy substrates utilised in the same period.

### Statistical analysis

All data are presented as mean value ± SD. Student’s T-test for repeated measurements was used to assess significance of US intensity differences between GL and ST within each condition, and between conditions for each muscle. Regression line equations were calculated using the least square model. *P* values less than 0.05 were considered statistically significant. All statistical procedures were performed using IBM SPSS Statistics package (v. 25 for MacOs).

## Abbreviations

US: Ultrasound
GL: *Gluteus medius* muscle
ST: *Semitendinosus* muscle
HR: Heart rate
O_2_: Oxygen
V’O_2_: Oxygen consumption
